# Prevalence of Tobacco Use Among the Children in the Age Group of 13-15 Years in Sikkim After 5 Years of Prohibitory Legislation

**DOI:** 10.4103/0970-0218.40884

**Published:** 2008-04

**Authors:** JP Majra, Junita Basnet

**Affiliations:** Department of Community Medicine, K.S. Hegde Medical Academy, Mangalore, Karnataka, India; 1 Department of UHC Namnang, SMIMS, Tadong, Sikkim

## Introduction

Despite increasing awareness to the harmful effects of tobacco, the use of tobacco in various forms continues to be a significant health risk factor. It has been predicted by the World Health Organization (WHO) that more than 500 million people alive today will be killed by tobacco use by 2030 and tobacco consumption will become the single leading cause of death.(1) In India, it is estimated that on current trends tobacco will kill 80 million males currently aged 0-34 years.(2) Considering the social and economic impact of tobacco consumption, Government of India introduced “Cigarettes and other tobacco products (Prohibition of Advertisement and regulation of Trade and Commerce, Production, Supply and Distribution) Bill 2001,” which was enacted in 2003. The state of Sikkim imposed a ban on the advertisement of tobacco in any form and sale of cigarettes to the minors and smokeless tobacco as a whole in the state since July 2001. The Tobacco Free Initiative of World Health Organization in collaboration with the Office on Smoking and Health, Centers for Disease Control, USA has undertaken Global Youth Tobacco Survey (GYTS) in Sikkim during January-March 2001 as in the rest of India, which revealed that 54.7% of the children between 13 and 15 years of age are using tobacco in one form or the other(3). The present study was carried to assess the effectiveness of the existing legislation to control tobacco use.

## Materials and Methods

The study was conducted in the east district of Sikkim in India in the year 2006. A written permission to conduct this study among the school children was obtained from the Human Resource Development Department, Government of Sikkim. The study group of 13 to 15 years students corresponds to grade VIII-X. A list of schools (both government and private) having grade VIII-X was obtained. Ten schools were selected randomly from the list and all the students in classes VIII to X were included in the study. Absence from the school on the day of study was taken as non-response. A pre-tested self-administered multiple choice questionnaire was used for data collection. A verbal consent was obtained from the principals of the schools and the students. Then, all the questions were explained to the students and total confidentiality was assured. Class teachers were requested to stay away while questionnaire was introduced. The tobacco products were categorized into two groups: smoking and smokeless, i.e. non-smoking tobacco products such as pan masala, gutka, khaini, mishri, etc. Data obtained were complied and analyzed using appropriate statistical methods. Chi-square test was applied to find out the statistical significance.

## Results

The present study represents 8000 students studying in 92 schools. The sample size was 1012 students in 10 schools. The response rate was 100% for schools and 81.4% for the students. Absence from the school on the day of study was the only cause for non-response. Among the respondents, 50.1% were boys. Majority (54%) were Hindus and 34.5% were Buddhists. More than 84% of the respondents were aware about various tobacco products available in the market. Table 1 shows the prevalence of tobacco use among the children in the age group of 13-15 years. Tobacco use among males was found to be significantly high (42.1%) when compared to that of females (17.0%). Cigarette smoking was more fashionable among boys (28.6%) and smokeless tobacco use was common amongst the girls (11.9%).

**Table 1:** Prevalence of tobacco use among the children in the age group of 13-15 years

	Tobacco-users				Non-users	Total
			
Cigarette	Smokeless	Both	Total		
Male	118 (28.6)	88 (19.1)	32 (7.8)	174 (42.1)	239 (57.9)	413 (100) [50.1]
Female	25 (6.1)	49 (11.9)	4 (1.0)	70 (17.0)	341 (83.0)	411 (100) [49.9]
Total	143 (17.4)	137 (16.6)	36 (4.37)	244 (29.6)	580 (70.4)	824 (100) [100]

A statistically significant decline in the overall use of tobacco [Table 2] is observed among the children in the age group of 13-15 years, which is mainly due to a decline in the use of smokeless tobacco; however, cigarette smoking has rather shown an increase (from 24.1% to 28.6%), although statistically insignificant, among the males.

**Table 2:** Change in the prevalence of tobacco use over the past 5 years (2001-2006)

Tobacco product	GYTS (2001)(4)	Present study (2006)	*P*-value
Cigarette			
Males	24.1	28.6	>0.05
Females	10.5	6.1	>0.05
Total	18.0	17.4	>0.05
Smokeless tobacco			
Males	45.5	19.1	<0.05
Females	28.6	11.9	<0.05
Total	37.9	16.6	<0.05
All forms			
Males	68.1	42.1	<0.05
Females	38.3	17.0	<0.05
Total	54.6	29.6	<0.05

The use of various forms of tobacco by family members/friends was the most important motivating factor among the users (65.6%), followed by actors using it on television or in movies (26.4%) and advertisement in the print/video media (8.3%). More than 70% of the family members and more than 74% of the friends of the users were using tobacco in some form in comparison to 55% of the family members and 41.4% friends for the non-users. Surprisingly, 36% of the users bought the product themselves for their first-time use and 57.9% got it from the friends and 5.3% from the family members. More than 92% respondents were aware about the harmful effects of the tobacco and over 65% expressed their desire to quit.

However, 244 (100%) of the users and 219 (37.8%) of the non-users were having a positive attitude towards tobacco use, thus exposing them to the risk of tobacco use. Eighty-nine (36.5%) of the users and 80 (13.8%) of the non-users feel that tobacco users look more attractive, and 42 (17.2%) of the users and 37 (6.4%) of the non-users think that tobacco users make more friends. One hundred and twenty-two (50%) of the users and 102 (17.6%) of the non-users had a wrong belief that tobacco users are benefited by the way of relief from stress, toothache, constipation, etc.

## Discussion

Tobacco is the second major cause of death in the world and the fourth most common risk factor for disease worldwide.(5) However, it is an irony that the tobacco use is the preventable cause of the death. It is amenable to simple cost-effective interventions. The present study clearly shows that the legislations are effective to control the menace of tobacco use to the extent that they are implemented and enforced strictly. It is observed that only 8.3% of the students consider the advertisement in print/video media, as a motivating factor compared to 65.6% family members/friends using tobacco and 26.4% actors using it on television or in movies. This may be due to limited exposure to advertisements in media as there is total and effective ban on advertising tobacco in any form in local media (personal observation). But the fact remains that in spite of total ban on the sale of smokeless tobacco products, these are available although not as easily as cigarettes (personal observation), impact of which is reflected in trends in the tobacco use. A statistically significant decline is observed in the use of smokeless tobacco only. Furthermore, as a large proportion (29.6%) of the children in the tender age group of 13-15 years were induced to the habit of tobacco use in the past 5 years. The study also reveals the presence of motivating factors such as social acceptance and positive attitude among the users as well as non-users towards the tobacco use.

## Conclusion and Recommendations

Law can prove to be useful in curbing the tobacco epidemic to the extent it is implemented and enforced. Therefore, to curb the tobacco use menace in the presence of motivating factors such as social acceptance and positive attitude towards tobacco use, a comprehensive approach is needed that also addresses the issues such as misconceptions in the society regarding tobacco, creating a supportive environment by changing social norms and medical support to treat nicotine dependence for those who wish to quit tobacco, in addition to the stringent legislation implemented in word and spirit.
